# Data on effect of Sacubitril/valsartan on cardiac Remodelling in diabetic Cardiomyopathy rats

**DOI:** 10.1016/j.dib.2021.106963

**Published:** 2021-03-18

**Authors:** Jiao Ai, Jing Liu, Mao Liu

**Affiliations:** Department of Internal Medicine, Affiliated Hospital of North Sichuan Medical College, China

**Keywords:** Diabetic cardiomyopathy, Sacubitril/valsartan, Cardiac remodeling

## Abstract

Diabetic cardiomyopathy (DCM) is one of the most important causes of death in patients with diabetic mellitus. The effect of sacubitril/valsartan (Sac/Val) on cardiac remodeling in DCM remains unclear. The present raw data were analysed to assess the effect of Sac/Val-on cardiac remodeling and explore the mechanisms of Sac/Val-in protecting the myocardium in DCM rats. The data include the left ventricular/body mass index, IL-6 and TNF-α levels in myocardial tissue, the protein expression levels of phos-p38, Col Ⅰ and Col Ⅲ in myocardial tissue between pre- and post- Sac/Val-treatment in DCM rats. Compared to DCM rats, the degree of fibrosis and the protein expression of phos-p38, Col Ⅰ and Col Ⅲ reduced after the treatment of Sac/Val. These data may provide useful bases for future clinical research concerning the application of Sac/Val-in patients with DCM.

## Specifications Table

**Table:** 

Subject	Medicine and Dentistry
Specific subject area	Cardiovascular Disease
Type of data	Figures
How data were acquired	Rat model of diabetic cardiomyopathy EILISA kit Western Blot SPSS 20.0 software
Data format	Raw Analysed
Parameters for data collection	ELISA kit was used to detect IL-6 and TNF-α level in myocardial tissue. Western Blot was used to detect the protein expression of p38, phos-p38, Col Ⅰ and Col Ⅲ in myocardial tissues of DCM rats.
Description of data collection	The body weight was measured before the diabetic cardiomyopathy rats were sacrificed. The left ventricular weight was weighed with an electronic balance. The content of IL-6 and TNF-α in the supernatant of myocardial tissue was measured with ELISA kits. Western Blot was used to detect the protein expression levels of p38, phos-p38, Col Ⅰ and Col Ⅲ in myocardial tissues of rats in each group. All data were analysed using SPSS 20.0 statistical software.
Data source location	Nanchong, China
Data accessibility	Repository name: [Sac/Val-in Diabetic Cardiomyopathy] Direct URL to data: http://dx.doi.org/10.17632/cn59ykrrvy.2

## Value of the Data

• The presented data are useful for expanding the indications of sacubitril/valsartan in the treatment of diabetic cardiomyopathy.
• These data will be of benefit to researchers who are interested specifically in the field of diabetic cardiomyopathy and heart failure.
• These data may be used as a basic research support and open avenues for further clinical trials on the application of sacubitril/valsartan in patients with diabetic cardiomyopathy.

## 1 Data Description

**Fig. 1:**
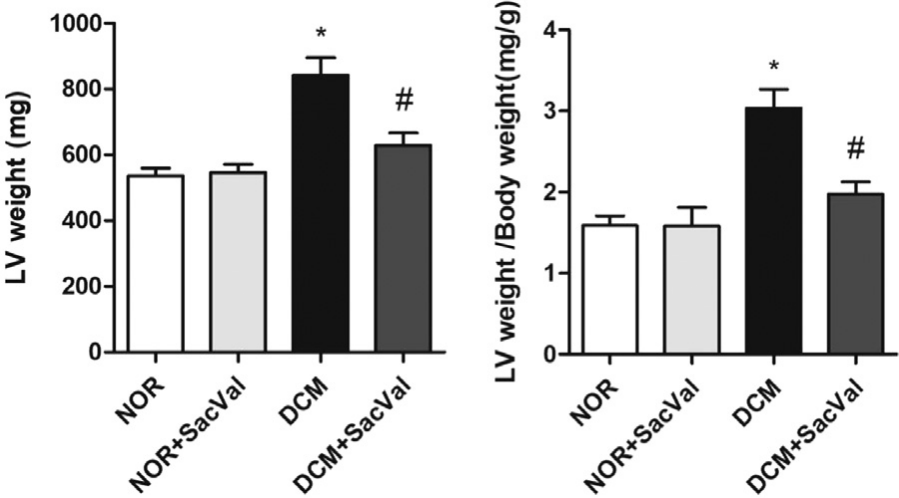
Effect of Sac/Val-on ventricular remodeling in DCM rats. LV, left ventricle; NOR, normal group; Sac/Val, sacubitril/valsartan; DCM, diabetic cardiomyopathy; *Compared to NOR group, *P*<0.05; ^#^Compared to DCM group, *P*<0.05.

**Fig. 2:**
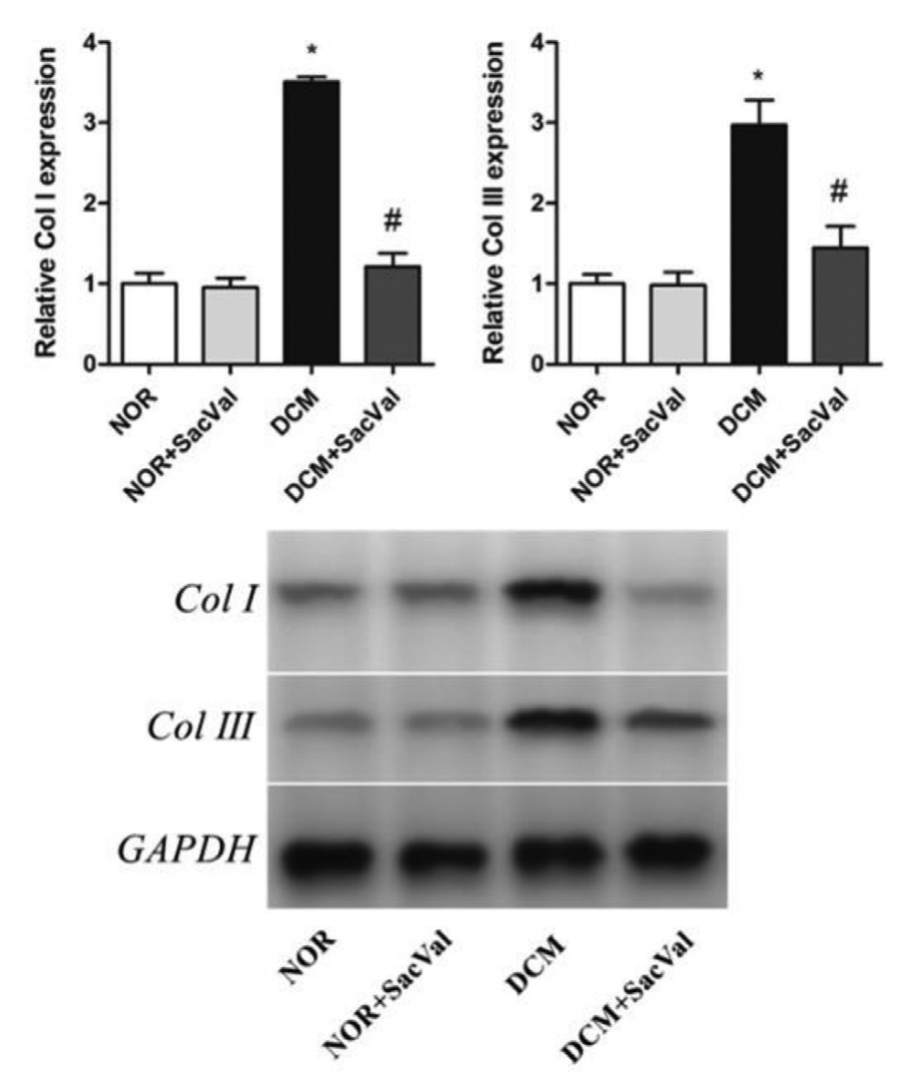
Effect of Sac/Val-on collagen expression in DCM rats. Col, collagen; NOR, normal group; Sac/Val, sacubitril/valsartan; DCM, diabetic cardiomyopathy; *Compared to NOR group, *P*<0.05; ^#^Compared to DCM group, *P*<0.05.

**Fig. 3:**
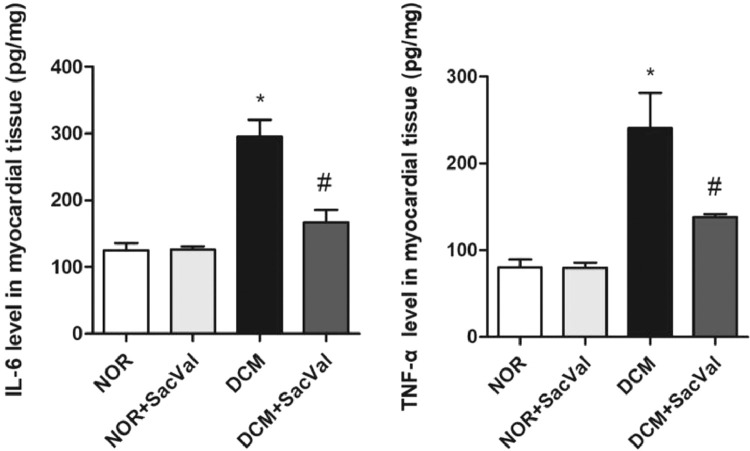
Effect of Sac/Val-on inflammatory factors in myocardial tissue in DCM rats. IL-6, interleukin 6; TNF-α, tumor necrosis factor α; NOR, normal group; Sac/Val, sacubitril/valsartan; DCM, diabetic cardiomyopathy; *Compared to NOR group, *P*<0.05; ^#^Compared to DCM group, *P*<0.05.

**Fig. 4:**
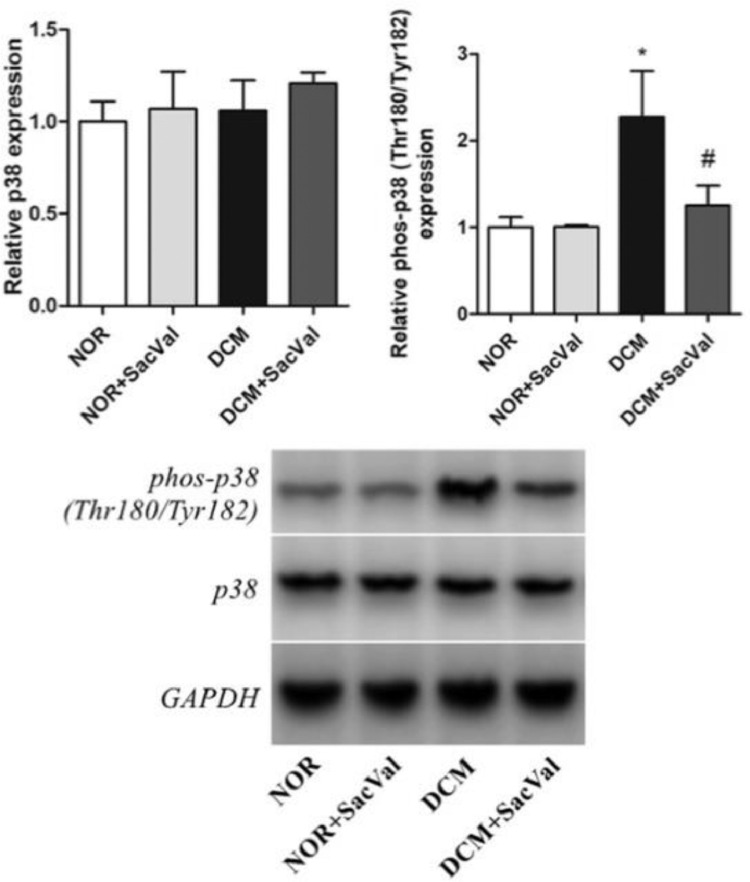
Effect of Sac/Val-on relative p38 and phos-p38 protein expression in myocardial tissue in DCM rats. NOR, normal group; Sac/Val, sacubitril/valsartan; DCM, diabetic cardiomyopathy; *Compared to NOR group, *P*<0.05; ^#^Compared to DCM group, *P*<0.05.

Diabetic cardiomyopathy (DCM) refers to a cardiac dysfunction observed in patients with diabetes that occurs in absence of other cardiovascular disease, such as coronary artery disease, hypertension and congenital heart disease [1]. In recent years, several important studies in the field of heart failure with reduced ejection fraction have shown that sacubitril/valsartan (Sac/Val) is superior to traditional drugs angiotensin-converting enzyme inhibitors /angiotensin Ⅱ blockers [2]. The present data provide evidence for the treatment of DCM by Sac/Val-in a rat model. Each group consists of five male Wistar rats. By comparing the differences in protein expression of p38, phos-p38, Col Ⅰ and Col Ⅲ in myocardial tissues in each group, we report the effect of Sac/Val-in alleviating cardiac remodeling in DCM rats. Fig. 1 describes the LVW/BW ratios in different groups. Fig. 2 describes the relative expression of Col Ⅰ and Col Ⅲ protein in each group. Fig. 3 describes the levels of inflammatory factors IL-6 and TNF-α in the four groups. Fig. 4 describes the expression of phos-p38 and p38 protein in each group.

## 2 Experimental Design, Materials and Methods

### 2.1 Materials

Twenty 8-week-old male Wistar rats were provided by the Animal Model Center of Nanjing University and the animal certificate number was SCXK (Su) 2015–0001. Streptozotocin (Sigma, St. Louis, MO, USA). Sacubitril/Valsartan (Sac/Val) Sodium Tablets (Novartis, Switzerland). Rat IL-6 ELISA Kit (ab100772), Rat TNF-α ELISA kit (ab100785), Anti-p38 antibody (ab31828), Anti-phos-p38 (Thr180/Tyr182) antibody (ab4822), Anti-Col I Antibody (ab6308) and Anti-Col III antibody (ab184993) were purchased from Abcam (Abcam, Cambridge, UK).

### 2.2 Model building

One-time intraperitoneal injection of 1% STZ (pH=4.5, sodium citrate buffer at 4 °C) at a dose of 70 mg/kg destroyed the function of pancreatic islet β cells. The tail venous blood of the rats was taken on the 3rd and 7th day after streptozotocin injection to detect the fasting blood glucose of the rats (fasting for 8 h). When both blood glucose were ≥16.7 mmol/L, and the rats had polydipsia, polyphagia, and polyuria. Then a rat model of diabetic cardiomyopathy has been established.

### 2.3 Grouping

The rats were divided into the following four groups: 1) Normal rats group (NOR): Without modeling operation, rats were given physiological saline (1 ml/100 g/d) by gavage for 8 weeks. 2) NOR + Sac/Val-group: Without modeling operation, rats were given Sac/Val (60 mg/kg/d) by gavage for 8 weeks; 3) DCM model group (DCM): After establishing the model, rats were given normal saline (1 ml/100 g/d) by gavage for 8 weeks. 4) DCM + Sac/Val-group: After modeling, rats were given Sac/Val (60 mg/kg/d) by gavage for 8 weeks.

### 2.4 Laboratory examination

The body weight (BW) was measured before the rats were sacrificed. Immediately removed the heart, freed the left ventricle, blotted it dry with filter paper, and weighed the left ventricular weight (LVW) with an electronic balance. LVW/BW (mg/g) = left ventricle weight (mg)/body weight (g). The content of IL-6 and TNF-α in the supernatant of myocardial tissue was measured with an ELISA kit. Western Blot was used to detect the protein expression levels of p38, phos-p38, Col Ⅰ and Col Ⅲ in myocardial tissues of rats in each group.

### 2.5 Statistical analysis

ANOVA one-way analysis of variance was used to compare the means of multiple independent samples. Least-significant difference (LSD) method was chosen as the post-hoc *t*-test. If there is uneven variance or non-normal distribution, the Kruskal-Wallis test is used for analysis. The significance level is bilateral α=0.05. All data were analyzed using SPSS 20.0 statistical software.

## Ethics Statement

This study was based on the Helsinki Declaration and the guidelines for the care and use of laboratory animals and approved by local ethics committee (Ethics approval number: 2020ER133–1).

## CRediT Author Statement

**Jiao Ai:** Methodology, Investigation, Resources, Data Curation, Writing - Original Draft; **Jing Liu:** Software, Formal analysis, Investigation, Resources; **Mao Liu:** Conceptualization, Methodology, Data Curation, Writing - Review & Editing, Supervision, Funding acquisition.

## Declaration of Competing Interest

The authors declare that they have no known competing financial interests or personal relationships, which have or could be perceived to have influenced the work reported in this article.
